# Incidence of Guillain–Barré syndrome in South Korea during the early COVID-19 pandemic

**DOI:** 10.3389/fneur.2023.1125455

**Published:** 2023-02-21

**Authors:** Sun Ah Choi, Junho Hwang, Byung Chan Lim, Soo Ahn Chae

**Affiliations:** ^1^Department of Pediatrics, Ewha Womans University Mokdong Hospital, Ewha Womans University College of Medicine, Seoul, Republic of Korea; ^2^Department of Pediatrics, Chung-Ang University Hospital, Chung-Ang University College of Medicine, Seoul, Republic of Korea; ^3^Department of Pediatrics, Seoul National University Children's Hospital, Seoul National University College of Medicine, Seoul, Republic of Korea

**Keywords:** Guillain-Barré syndrome, SARS-CoV-2, COVID-19, nationwide infection, *Campylobacter*

## Abstract

**Objectives:**

Guillain–Barré syndrome (GBS) is an immune-mediated polyradiculoneuropathy, often triggered by infection. We aimed to investigate how the incidence of GBS changed in the early stages of the coronavirus 2019 (COVID-19) pandemic when nationwide infections declined due to non-pharmaceutical interventions.

**Methods:**

We conducted a nationwide population-based retrospective GBS cohort study using data from the Health Insurance Review and Assessment Service of Korea. Patients with new-onset GBS were defined as those who were first hospitalized between 1 January 2016 and 31 December 2020 with an International Classification of Disease, 10th Revision code, for GBS (G61.0) as a primary diagnosis. The incidence of GBS in the pre-pandemic years (2016–2019) was compared with that in the first pandemic year (2020). Nationwide epidemiological data for infections were collected from the national infectious disease surveillance system. A correlation analysis was performed to determine the incidence of GBS and nationwide trends of various infections.

**Results:**

Overall, 3,637 new-onset GBS cases were identified. The age-standardized incidence of GBS in the first pandemic year was 1.10 (95% confidence interval, 1.01–1.19) per 100,000 persons. Compared to the first pandemic year, the incidence of GBS during the pre-pandemic years (1.33–1.68/100,000 persons/year) was significantly higher, with incidence rate ratios of 1.21–1.53 (*P* < 0.001). Nationwide cases of upper respiratory viral infections were significantly reduced in the first pandemic year; however, *Campylobacter* infections peaked in the summer of the pandemic. The nationwide epidemiology of parainfluenza virus, enterovirus, and *Campylobacter* infections correlated positively with GBS incidence.

**Conclusion:**

The overall GBS incidence decreased in the early stages of the COVID-19 pandemic, which can be attributed to the dramatic reduction in viral illnesses due to public measures.

## 1. Introduction

Guillain–Barré syndrome (GBS) is an acute, post-infectious, immune-mediated polyradiculoneuropathy characterized by progressive motor weakness, areflexia or hyporeflexia, and sensory deficits. Approximately two-thirds of the patients report symptoms of respiratory or gastrointestinal infections before the onset of neurological symptoms (1). Several infectious pathogens, including *Campylobacter jejuni*, cytomegalovirus, Epstein–Barr virus, *Mycoplasma pneumoniae*, and Zika virus, have been reported as triggering factors (1–4). The pathological mechanism of GBS triggered by infection has often been explained by molecular mimicry of antibodies against human peripheral nerve gangliosides (5, 6).

The incidence of GBS in relation to severe acute respiratory syndrome coronavirus type 2 (SARS-CoV-2) infection remains controversial. Several case series have reported that the GBS incidence has increased since SARS-CoV-2 acts as a potential triggering factor for GBS (7–10). GBS has been reported as a neurological manifestation following SARS-CoV-2 infection (11, 12). The pathogenesis of GBS after SARS-CoV-2 infection is suggested to be immune-mediated, rather than a direct invasion of the nervous system (13). Conversely, other studies have reported no increase in GBS incidence during the early coronavirus disease 2019 (COVID-19) pandemic. In the early stages of the COVID-19 pandemic, non-pharmaceutical interventions (NPIs), such as social distancing, avoiding physical contact and mass gatherings, and universal use of facemasks, were implemented to control the spread of COVID-19. These NPIs have lowered the infection rates of acute respiratory viruses, mumps, measles, and gastrointestinal viral illnesses, which could trigger GBS (14, 15).

The primary aim of this study was to demonstrate how the incidence of GBS changed in the first COVID-19 pandemic year (2020) compared to that in the pre-pandemic years (2016–2019). We investigated the incidence of GBS before and during the COVID-19 pandemic in a nationwide population-based retrospective GBS cohort. The secondary aim was to demonstrate how GBS incidence changed in relation to the epidemiology of nationwide infections.

## 2. Methods

### 2.1. Data sources

We conducted a nationwide population-based retrospective GBS cohort study using the Health Insurance Review and Assessment Service (HIRA) database (dataset No. M20210210108). The HIRA database consists of claims from ~98% of the Korean population (nearly 50 million individuals) and includes data on demographics, diagnoses, prescriptions, procedures, and medical costs (16). Diagnostic codes were assigned based on the Korean Classification of Diseases, seventh version (KCD-7), which is a modified version of the International Classification of Diseases, 10th Revision (ICD-10).

For the epidemiologic data on infectious diseases, we used national statistics from the national infectious disease surveillance system operated by the Korea Disease Control and Prevention Agency (KDCA). The surveillance system demonstrates nationwide trends in notifiable infectious diseases according to the identified pathogens collected from more than 200 sentinel healthcare centers in Korea (17). These data are available to the public on the KDCA infectious disease portal website (https://www.kdca.go.kr/npt), without copyright issues.

### 2.2. Study population

We defined new-onset GBS as the first hospitalization (ICD-10 code G61.0) with a primary diagnosis from 1 January 2016 to 31 December 2020 (18–20). GBS was defined as severe when the patients with first primary-coded GBS hospitalization required an intravenous immunoglobulin (IVIg) prescription for at least 3 days. Data on the following variables of patients with GBS were extracted from the HIRA database: age, sex, medications used, length of hospital admission, use of intensive care unit (ICU) care, and use of invasive mechanical ventilators during admission.

### 2.3. Epidemiologic data of infectious diseases

We selected several notifiable infectious pathogens, including parainfluenza virus, respiratory syncytial virus, rhinovirus, adenovirus, influenza-like illness, enterovirus, *Campylobacter*, and *Salmonella*, from the national infectious disease surveillance system. We collected weekly and monthly data for these pathogens from 1 January 2016 to 31 December 2020.

### 2.4. Statistical analysis

The baseline characteristics of new-onset GBS cases are presented either as means with standard deviations or as frequencies. The age-standardized incidence of GBS was calculated based on the corresponding mid-year population obtained from the Korean Statistical Information Service. Pairwise comparisons between years were evaluated using incidence rate ratios (IRRs) and the corresponding 95% confidence intervals (CIs). The student's *t*-test was used to determine the statistical significance between each annual incidence and the first pandemic year. We visualized the trends of GBS incidence and infectious diseases using time-series analysis and locally weighted scatterplot smoothing (LOWESS). We constructed a seasonal autoregressive integrated moving average (ARIMA) model to forecast the incidence of GBS in the first pandemic year based on data from the pre-pandemic years. The parameters were determined by comparing multiple candidate models in terms of residuals, autocorrelation coefficients, and the Ljung–Box test. The observed incidences and model predictions were examined to determine whether the observed incidences after the COVID-19 outbreak were within the 95% CIs of the predicted values. Pearson's correlation analysis was performed to determine the relationship between GBS incidence and nationwide infection trends. *P* < 0.05 was considered to indicate statistical significance. Statistical analyses were performed using R version 4.0.5 (R Foundation for Statistical Computing, Vienna, Austria) and SAS (version 9.4; SAS Institute, Cary, NC, USA).

## 3. Results

### 3.1. Incidence of GBS

A total of 3,637 patients with incident GBS were identified. Among them, 1,488 had severe GBS and required IVIg therapy for at least 3 days (Table 1). Of the 3,637 GBS cases, 13.9% were admitted to the ICU and 10.0% required invasive mechanical ventilation. Compared to the pre-pandemic years, the proportion of GBS cases requiring IVIg therapy, ICU care, and invasive mechanical ventilation did not increase during the pandemic.

**Table 1:** Characteristics of patients with GBS.

**Characteristics**	**2016**	**2017**	**2018**	**2019**	**2020**
GBS cases, *n*	779	758	850	680	570
Age, years (±SD)	51.1 (±21.0)	49.5 (±20.5)	50.3 (±21.1)	53.5 (±21.3)	53.0 (±20.4)
Male:female, *n*	459:320	469:289	525:325	388:292	363:207
Severe GBS cases, *n* (%)	263 (33.8)	295 (38.9)	366 (43.1)	296 (43.5)	268 (47.0)
Length of hospital stay, days (±SD)	47.3 (±123.4)	42.3 (±84.5)	41.4 (±87.3)	39.0 (±70.8)	28.1 (±39.4)
ICU care, *n* (%)	112 (14.4)	95 (12.5)	125 (14.7)	95 (14.0)	78 (13.7)
Invasive mechanical ventilation during illness, *n* (%)	78 (10.0)	75 (9.9)	83 (9.7)	76 (11.2)	53 (9.3)

GBS, Guillain–Barré syndrome; SD, standard deviation; ICU, intensive care unit.

**Figure 1:**
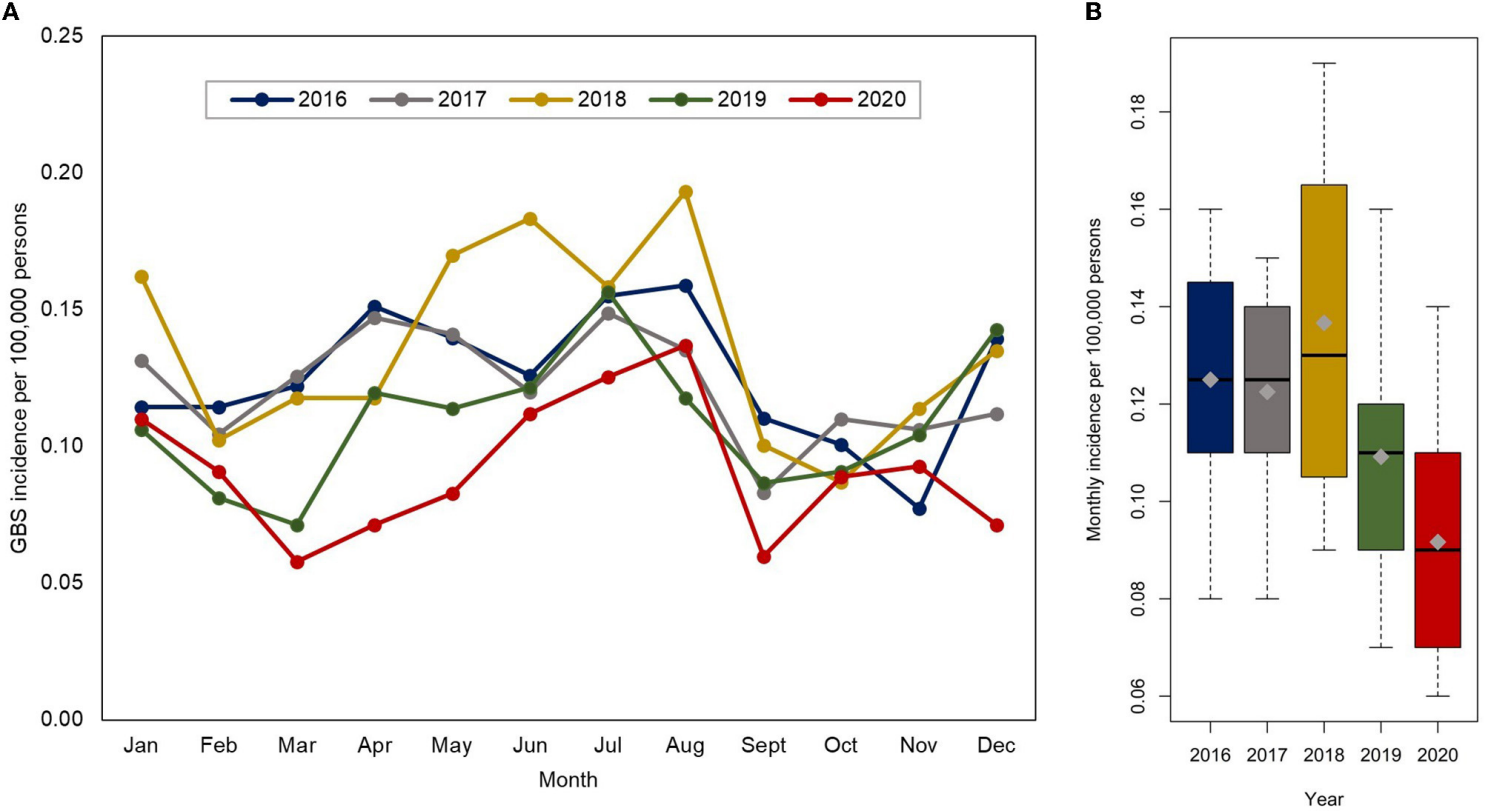
**(A)** Monthly incidence of GBS per 100,000 persons in each year (2016–2020). **(B)** Boxplots presenting the monthly incidence of GBS each year. GBS, Guillain–Barré syndrome.

**Figure 2:**
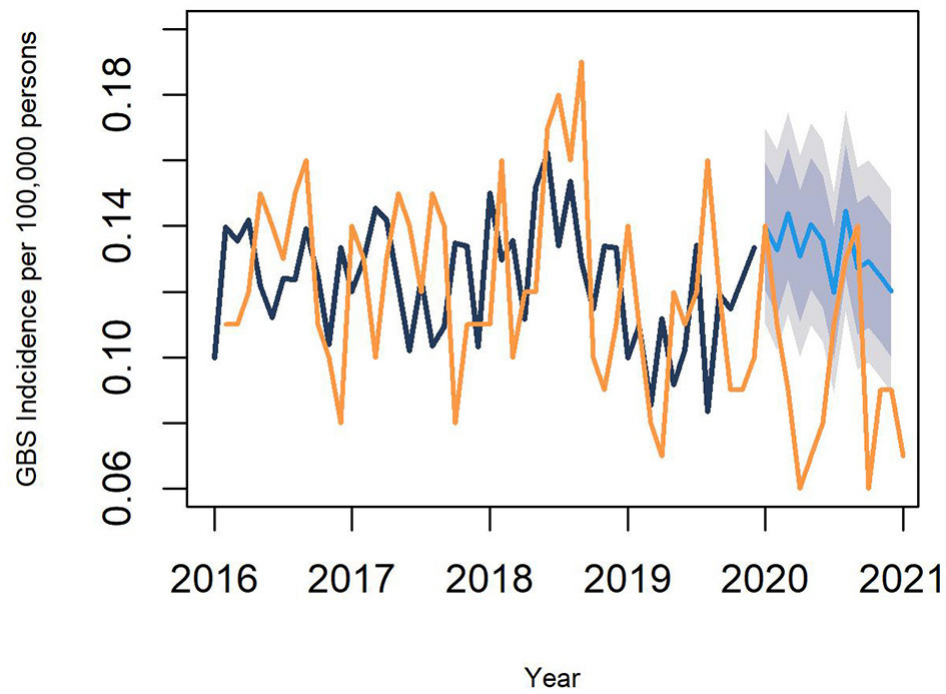
Incidence of GBS predicted by an autoregressive integrated moving average model. The orange line denotes the observed incidence and the black line is fitted by removing a seasonal variation from the observed incidence. The blue line denotes the predicted incidence during the COVID-19 pandemic, and the blue shades represent 80 and 95% CIs of the predicted incidence during the pandemic. GBS, Guillain–Barré syndrome.

The age-standardized incidence of GBS in the pre-pandemic years was 1.33–1.68 per 100,000 persons per year (Table 2). The age-standardized incidence of GBS in the first pandemic year dropped to 1.10 (95% CI, 1.01–1.19) per 100,000 persons. Compared to the first pandemic year, the age-standardized incidence of GBS during the pre-pandemic years was significantly higher, with IRRs of 1.21–1.46 (*P* < 0.001). In both the pre-pandemic years and the first pandemic year, there was a significant seasonal peak in the incidence in summer (Figure 1). Despite a decline in the overall GBS incidence in the first pandemic year, there was still seasonal variation that peaked from June to August. This summer peak lies within the 95% CIs of the incidence predicted by the ARIMA model, whereas the overall incidence observed during the pandemic was lower than the predicted values (Figure 2).

**Table 2:** Age-standardized incidence and incidence rate ratio of GBS during 2016–2020.

		**IRR (95% CI)**	
**Year**	**Age-standardized incidence per 100,000 persons (95% CI)**	**Crude IRR**	**Age-adjusted IRR**
2016	1.60 (1.49–1.71)	1.37(1.23–1.52)	1.46(1.31–1.62)
2017	1.52 (1.41–1.62)	1.33(1.20–1.49)	1.38(1.24–1.54)
2018	1.68 (1.57–1.79)	1.49(1.34–1.66)	1.53(1.38–1.70)
2019	1.33 (1.23–1.43)	1.19(1.07–1.33)	1.21(1.08–1.35)
2020	1.10 (1.01–1.19)	Reference	Reference

GBS, Guillain–Barré syndrome; IRR, incidence rate ratio; CI, confidence interval.

### 3.2. Epidemiology of nationwide infections

**Figure 3:**
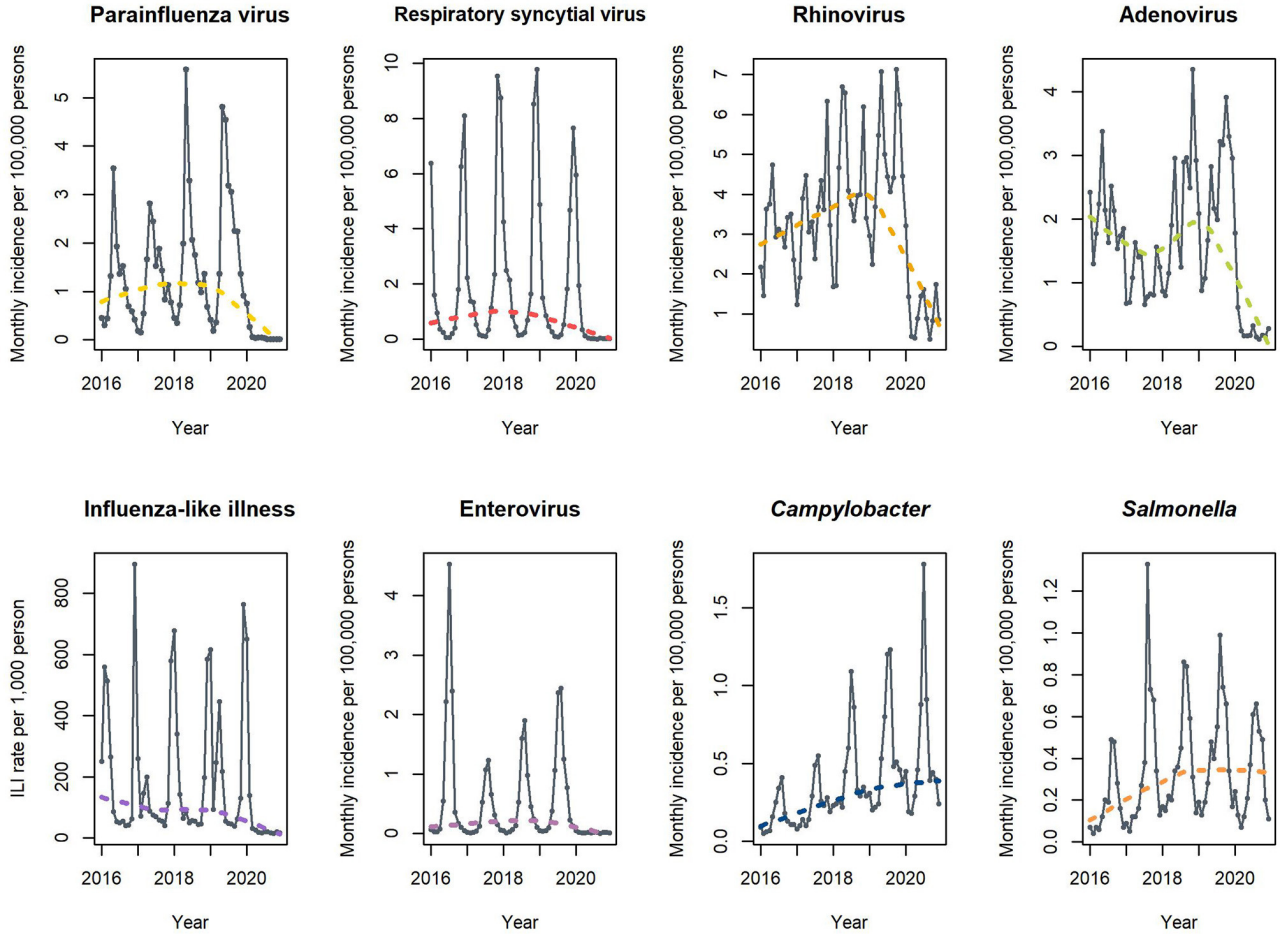
Time-series analysis of the nationwide infections from a nationwide infectious surveillance system. Dotted lines present the trends of infectious diseases using locally weighted scatterplot smoothing. ILI, Influenza-like illness.

In the pre-pandemic years, there was a remarkable seasonal variation in each infectious disease in the time-series analysis (Figure 3). However, in the first pandemic year, the time series as well as LOWESS demonstrated a significant decline in nationwide viral infections with a loss of seasonal variation. Conversely, the incidence of *Campylobacter* and *Salmonella* infections did not decrease in the first pandemic year, and their incidences were high during the first summer of the pandemic.

### 3.3. Correlation between GBS incidence and the trends of nationwide infections

**Figure 4:**
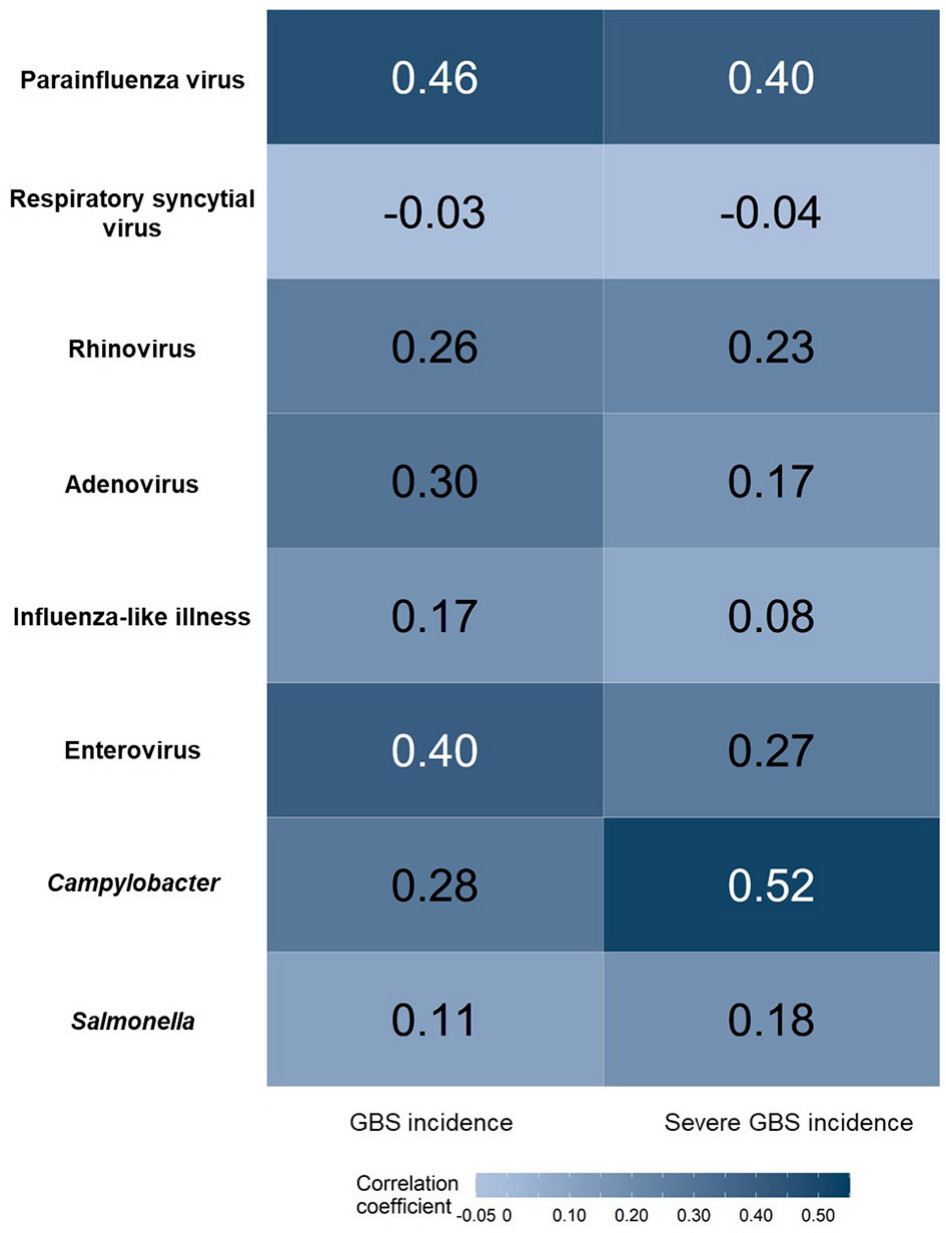
Correlation between GBS incidence and the trends of each infection. Positive correlations are shown in dark blue. GBS, Guillain–Barré syndrome.

The results of the correlation analysis between GBS incidence and the trends of each infection are presented in Figure 4 (and Supplementary Table 1 in detail). The nationwide epidemiology of parainfluenza virus, enterovirus, and *Campylobacter* is positively correlated with the overall GBS incidence. Parainfluenza virus and *Campylobacter* infections also positively correlated with severe GBS incidence.

## 4. Discussion

This study demonstrates that the overall incidence of GBS decreased in South Korea during the first year of the COVID-19 pandemic. As the pathogenesis of GBS is often explained by a post-infectious inflammatory response, a dramatic reduction in viral illnesses due to NPIs in the early stage of the pandemic could be a reason for the decline in GBS incidence. Previous studies validated the working definition of the first primary-coded GBS hospitalization as having a high positive predicted value (86% in a Danish nationwide study and 75% in a Korean nationwide study) (18, 19). The GBS incidence in the pre-pandemic years (between 1.33 and 1.68 cases/100,000 persons/year) in our study was similar to that reported in prior epidemiological studies (1.24–1.77 cases/100,000 persons/year), indicating the reliability of our results in terms of incidence (18, 20). We assessed the incidence of GBS and compared it with that in the pre-pandemic years using two methods. First, we measured the nationwide incidence of GBS and compared it before and during the pandemic. The number of new GBS cases and the monthly incidence of GBS in the first pandemic year decreased compared with those in the pre-pandemic years. Second, we compared the observed GBS incidence in the first year of the pandemic with that predicted by the ARIMA model. The observed monthly incidence of GBS in the first year of the pandemic was lower than the values predicted by the ARIMA model, except in summer. Our study confirmed that the overall incidence of GBS decreased in South Korea during the early stages of the COVID-19 pandemic.

However, there are conflicting reports on the incidence of GBS in relation to SARS-CoV-2 infection. A case series from Italy, Spain, and France reported an increase in GBS incidences from March to April 2020 (7–10). A study from Italy reported that the monthly incidence during March–April 2020 was 0.65 cases/100,000 persons per month, which is 5.4-fold higher than that during 2017–2019 (0.12 cases/100,000 persons per month) (9). The Spanish Emergency Network also reported that the frequency of GBS in patients with COVID-19 was higher than that in the non-COVID-19 population from March to April 2020 (10). These previous studies postulated the potential role of SARS-CoV-2 as a causative agent of GBS; however, they were performed on a small number of patients and the observation period was only the first few months of the ongoing pandemic. Conversely, an epidemiological study from the UK National Immunoglobulin Database between March and May 2020 reported the opposite result of a reduction in GBS incidence during the COVID-19 pandemic compared to that during 2016–2019 (21). They speculated that a decline in common transmissible infectious GBS triggers might explain the pandemic-related reduction of GBS cases. A subsequent study from the International GBS Outcome Study conducted between January and May 2020 also found no increase in the recruitment of patients with GBS during the pandemic (22). However, these studies did not provide information on how the epidemiology of common transmissible infections changed during the first year of the pandemic.

Nationwide infectious surveillance data for the first full year of the COVID-19 pandemic demonstrated different epidemiological patterns between viral infections and *Campylobacter* infections. Since the implementation of NPIs, most viral illnesses transmitted by direct contact or droplets decreased significantly during the pandemic (14). Unlike viral pathogens, *Campylobacter* and *Salmonella* spp. did not decrease during the COVID-19 pandemic (15). These bacterial pathogens are mainly transmitted *via* contaminated food, which is not affected by NPIs made for COVID-19. Similar to that reported in a study from northern China, the incidence of GBS and outbreak of *Campylobacter* infection shared epidemiological features, showing a seasonal peak in the summer, even in the pandemic year (5). *Campylobacter*, a GBS trigger, and viral infections were positively correlated with GBS incidence. This finding supports the notion that the GBS incidence in the first year of the COVID-19 pandemic could be influenced by a decrease in viral pathogens (21).

Our study had several limitations. COVID-19 can influence the incidence of GBS either by the infection itself or by changing patterns in healthcare utilization. A systematic review concluded that the risk of GBS was higher in patients with COVID-19 than in contemporary or historical controls (13). The COVID-19 outbreak in Korea was not as explosive as that in other countries in the early stage due to strict NPIs (Supplementary Figure 1). The incidence of GBS can vary when the COVID-19 infection increases dramatically. In this study, we could not analyze the risk of GBS according to the SARS-CoV-2 test results, as these were considered confidential at the time of analysis. Additional studies are required to confirm the potential causal relationship between SARS-CoV-2 infection and GBS. Patterns of healthcare utilization also changed during the pandemic, and general hospitalization as well as emergency and outpatient visits decreased, with a greater reduction in less severe illnesses (23, 24). The decline in GBS hospitalizations could be partly attributed to the overall reduction in healthcare utilization during the pandemic. However, we believe that an overall reduction in hospitalizations has a minimal impact on GBS hospitalizations. GBS is a rare neurological disease that requires specialized medical care owing to its acute and progressive nature. Approximately 90% of patients with GBS in our cohort were treated in general and tertiary healthcare centers, and healthcare utilization was unchanged during the pandemic (25). There are several limitations to the database studies. First, we were unable to ascertain the types of GBS variants, symptom severity, or other laboratory findings from the database. We adopted the working definition of new-onset GBS from previous GBS studies (18–20). Using the first hospitalization as the working definition, mild GBS cases that did not require hospitalization were not included in this study. In addition, the HIRA database and the national infectious disease surveillance system were not linked at the individual level; therefore, we could not analyze the association between infectious etiologies and GBS. Despite the limitations of the database study, the strengths of our study include the availability of nationwide longitudinal incidence with full coverage, minimizing selection bias, and allowing us to monitor the incidence of rare neurological diseases.

This nationwide retrospective cohort study revealed that the overall incidence of GBS decreased in South Korea during the first year of the COVID-19 pandemic. *Campylobacter* infection, which is usually transmitted by contaminated food, peaked during the first summer of the pandemic and positively correlated with GBS incidence. A dramatic reduction in viral illness due to NPIs in the early stages of the pandemic could have led to a decline in GBS incidence.

## Data availability statement

The original contributions presented in the study are included in the article/Supplementary material, further inquiries can be directed to the corresponding author.

## Ethics statement

The studies involving human participants were reviewed and approved by the Institutional Review Board of Chung-Ang University Hospital (IRB No. 2102-002-19352). Written informed consent from the patients/participants or patients/participants' legal guardian/next of kin was not required to participate in this study in accordance with the national legislation and the institutional requirements.

## Author contributions

SC designed the study, analyzed the data, wrote the paper, and provided funding for the study. JH collected, analyzed, and interpreted the data. BL and SC conceived the retrospective cohort design, interpreted the data, and revised the paper. All authors have read and approved the final version of the manuscript and made the decision to submit this manuscript.
