# Corrigendum: Incidence of Guillain-Barré syndrome in South Korea during the early COVID-19 pandemic

**DOI:** 10.3389/fneur.2023.1184177

**Published:** 2023-04-11

**Authors:** Sun Ah Choi, Junho Hwang, Byung Chan Lim, Soo Ahn Chae

**Affiliations:** ^1^Department of Pediatrics, Ewha Womans University Mokdong Hospital, Ewha Womans University College of Medicine, Seoul, Republic of Korea; ^2^Department of Pediatrics, Chung-Ang University Hospital, Chung-Ang University College of Medicine, Seoul, Republic of Korea; ^3^Department of Pediatrics, Seoul National University Children's Hospital, Seoul National University College of Medicine, Seoul, Republic of Korea

**Keywords:** Guillain-Barré syndrome, SARS-CoV-2, COVID-19, nationwide infection, *Campylobacter*

In the published article, there was an error in Figure 3 as published. Subheadings for each graph were missing. The corrected Figure 3 and its caption appear below.

**Figure 3:**
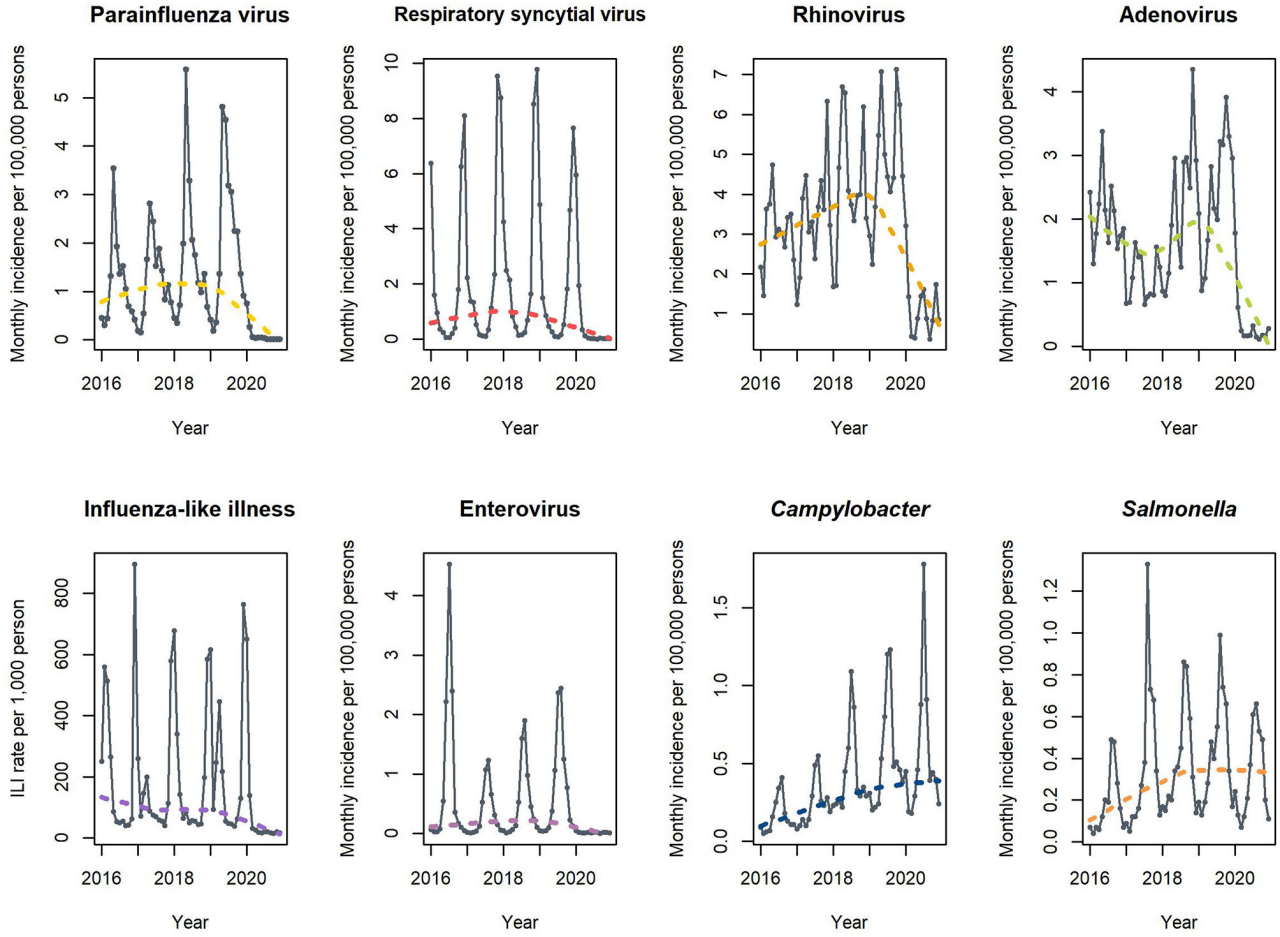
Time-series analysis of the nationwide infections from a nationwide infectious surveillance system. Dotted lines present the trends of infectious diseases using locally weighted scatterplot smoothing. ILI, Influenza-like illness.

The authors apologize for this error and state that this does not change the scientific conclusions of the article in any way. The original article has been updated.

