# Supplementary material for: Incidence of Guillain–Barré syndrome in South Korea during the early COVID-19 pandemic
Source: Front Neurol. 2023 Feb 21;14:1125455. doi: 10.3389/fneur.2023.1125455 (PMC9989167; doi:10.3389/fneur.2023.1125455)
Supplement: Supplementary file 1 [file Data_Sheet_1.docx]

Supplementary Material

Incidence of Guillain-Barré syndrome in South Korea
during the early COVID-19 pandemic

Sun Ah Choi,^1^ Junho Hwang,^2^ Byung Chan Lim,^3^ and Soo Ahn Chae^2*^

*** Correspondence:** Soo Ahn Chae, MD, PhD
Division of Pediatric Neurology, Department of Pediatrics
Chung-Ang University College of Medicine
Chung-Ang University Hospital
102, Heukseok-ro, Dongjak-gu, Seoul 06973, Republic of Korea
Tel.: +82 2-6299-1479
Fax: +82 2-6264-2167
E-mail: [kidbrain@cau.ac.kr](mailto:kidbrain@cau.ac.kr)

**Supplementary Table 1.** Correlation between GBS incidence and the nationwide infections.

|  | GBS | | Severe GBS | |
| --- | --- | --- | --- | --- |
|  | *r* | *P*-value | *r* | *P*-value |
| Parainfluenza virus | 0.46 | < 0.001 | 0.4 | 0.002 |
| Respiratory syncytial virus | -0.03 | 0.837 | -0.04 | 0.781 |
| Rhinovirus | 0.26 | 0.043 | 0.23 | 0.08 |
| Adenovirus | 0.3 | 0.022 | 0.17 | 0.193 |
| Influenza-like illness | 0.17 | 0.186 | 0.08 | 0.549 |
| Enterovirus | 0.4 | 0.002 | 0.27 | 0.034 |
| *Campylobacter* | 0.28 | 0.028 | 0.52 | < 0.001 |
| *Salmonella* | 0.11 | 0.385 | 0.18 | 0.167 |

GBS, Guillain-Barré syndrome

**Supplementary Figure 1.** Trends in the occurrence of COVID-19 from January 20, 2020 to December 31, 2020 in South Korea. COVID-19, coronavirus disease 2019


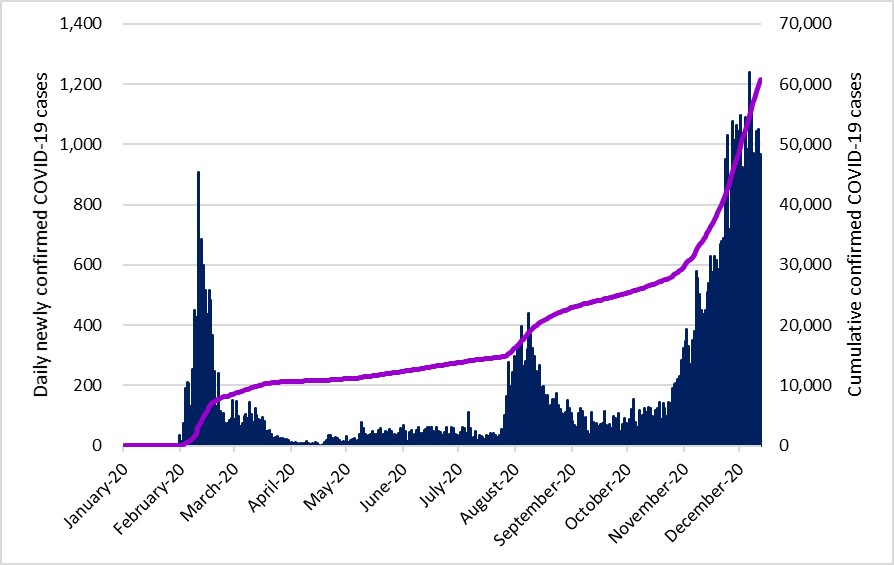


Source: Korea Ministry of Health and Welfare. Available online: <http://ncov.mohw.go.kr/> (accessed on October 1, 2022)
